# A Comprehensive Review on Nutritional Traits, Extraction Methods, Oxidative Stability, Encapsulation Technologies, Food Applications and Health Benefits of Omega Fatty Acids

**DOI:** 10.1002/fsn3.71008

**Published:** 2025-09-28

**Authors:** Muhammad Abdul Rahim, Malika Uzma, Fahad Al‐Asmari, Hyrije Koraqi, Mohamed Fawzy Ramadan, Roberto Castro‐Muñoz, Eliasse Zongo

**Affiliations:** ^1^ Department of Food Science & Nutrition, Faculty of Medicine and Allied Health Sciences Times University Multan Punjab Pakistan; ^2^ Department of Medical lab Technology Times University Multan Punjab Pakistan; ^3^ Department of Food and Nutrition Sciences, College of Agricultural and Food Sciences King Faisal University Al Hofuf Saudi Arabia; ^4^ Faculty of Food Science and Biotechnology UBT‐Higher Education Institution Prishtine Albania; ^5^ Department of Clinical Nutrition, Faculty of Applied Medical Sciences Umm Al‐Qura University Makkah Saudi Arabia; ^6^ Department of Sanitary Engineering, Faculty of Civil and Environmental Engineering Gdansk University of Technology Gdansk Poland; ^7^ Laboratory of Research and Teaching in Animal Health and Biotechnology Universite Nazi Boni Bobo‐Dioulasso Burkina Faso

**Keywords:** functional food, human health, microencapsulation, nutrition, oilseeds, plant seeds, technology

## Abstract

This review has studied omega fatty acids, their importance, functionality, health benefits, sources, oxidation, preservation, and the function of foods prepared with omega fatty acid edible sources. Omega fatty acids are essential polyunsaturated fatty acids (PUFAs), and their consumption has increased with increasing awareness about their health benefits. Plant oilseeds, including chia seeds (CS), flax seeds, linseeds, sesame seeds, and fish oil (FO), are some of the major sources of omega fatty acids. These essential omega fatty acids have been studied for their health benefits and effectiveness against the pathophysiology of various diseases, including metabolic dysfunction, cardiovascular diseases, malignancy, mental health, neurodegenerative diseases, liver diseases, systemic circulation, and infant mortality. Various in vitro and in vivo studies revealed their effectiveness against these diseases and their complications. Omega fatty acids also reduce the risk of these diseases by improving health and reducing risk factors. Omega fatty acids, being polyunsaturated, are highly susceptible to oxidation, which greatly affects their stability and affects their peroxide value at storage. Various preservation techniques, including traditional encapsulation, microencapsulation, and multilayer encapsulation, are studied and practiced to prevent oxidation. To make omega fatty acids bioavailable, various functional foods, including spreads, cookies, and other formulations, are prepared by food technologists, considering their physicochemical properties.

## Graphical abstract

This review has studied omega fatty acids, their importance, functionality, health benefits, sources, oxidation, preservation, and the function of foods. These essential omega fatty acids have been studied for their health benefits and effectiveness against the pathophysiology of various diseases. Various preservation techniques, including traditional encapsulation, microencapsulation, and multilayer encapsulation, are studied and practiced to prevent oxidation. To make omega fatty acids bioavailable, various functional foods, including spreads, cookies, and other formulations, are prepared by food technologists, considering their physicochemical properties.

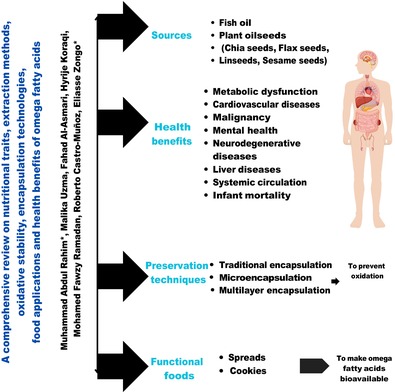

## 1 Omega Fatty Acids and Their Nutritional Importance

Long‐chain polyunsaturated fatty acids (LCPUFAs) consist of eicosapentaenoic acid (EPA) (20:5), docosahexaenoic acid (DHA) (22:6), stearidonic acid (18:4), and docosapentaenoic acid (22:5). Omega‐3 (Ω‐3) PUFAs consumption has increased in recent years due to their benefits to health. This work discusses omega fatty acid biochemistry, health benefits, including bioavailability, properties, feature structure, major food sources, metabolism process, heart diseases, chronic diseases, malignancy, brain diseases, as well as maternal and infant mortality risk. Although the potential health benefits of Ω‐3 PUFAs have been described in several research works, detailed in vivo and clinical studies of the safety and efficacy of these Ω‐3 PUFAs are needed (Shahidi and Ambigaipalan 2018). The effects of essential fatty acids, such as Ω‐3 and omega‐6 (Ω‐6) profiles [fish oil (FO), sunflower oil, and linseed oil], on animal liver were measured. The results show that a diet rich in liver phospholipid increases the arachidonic acid content with linoleic acid (LA), and the maximum amount of EPA and DHA in FO was reduced. The effect of linseed oil and FO increased the amount of EPA in liver phospholipids and increased DHA with the FO diet (Khadge et al. 2018; Fatima et al. 2019).

In Ω‐3 PUFAs, the double bond is placed on the 3rd carbon chain of the methyl group. Similarly, the double bond in Ω‐6 is placed on the 6th carbon chain of the methyl group (Watanabe and Tatsuno 2021). Elmadfa and Kornsteiner (2009) recommended that the dietary Ω‐6 PUFAs (LA) intake in the USA should be 17 g per day on average. Furthermore, ground nuts and vegetable oils are an excellent natural source of major Ω‐3 PUFAs (α‐linolenic acid, ALA) (Djuricic and Calder 2021). Long‐chain Ω‐3 fatty acids, including EPA and DHA, could also be manufactured from Ω‐3 essential fatty acids and have reduced enzyme performance (Ni et al. 2022). One of the most important structural roles of Ω‐3 essential fatty acids is as the key component in the cell membrane phospholipid. Two major families of long‐chain PUFAs (EPA and DHA) are present in the brain and retina's synaptic and other nerve membranes. Changes in the ratio of PUFAs structure affect the membrane width, flow, properties, and how integral membrane proteins are involved in motility and their functions. It is also suggested that the Ω‐3 essential fatty acid mediates the protection of powerhouse function and a significant reduction of neurodegenerative diseases (Öz et al. 2022). LA (18:2, Ω‐6) serves as the primary PUFAs prevalent in the majority of Western diets. It holds essential status as it cannot be endogenously synthesized in higher animals, including humans. The metabolic conversion of LA involves desaturation, leading to the formation of gamma‐linolenic acid (GLA; 18:3, Ω‐6), subsequent elongation resulting in dihomo‐gamma‐linolenic acid (20:3, Ω‐6), and a subsequent desaturation step to generate arachidonic acid (ARA). ARA, in turn, can undergo further metabolism to yield various Ω‐6 PUFAs (Khadge et al. 2018; Fatima et al. 2019). Olive oil, recognized for its elevated monounsaturated fatty acid content, particularly oleic acid {OA, C18:1, omega‐9 (Ω‐9)}, has conventionally been attributed as a key component of the Mediterranean diet, believed to confer beneficial effects. OA is classified within the Ω‐9 fatty acids family, encompassing fatty acids with their initial double bond at the Ω‐9 carbon. Diverging from Ω‐3 and Ω‐6 fatty acids, Ω‐9 fatty acids are deemed non‐essential due to the human capacity to synthesize OA from stearic acid, facilitated by Ω‐9‐desaturase. Typically, competition from C18:2, Ω‐6 and C18:3, Ω‐3 for Ω‐6 desaturase hinders the production and accumulation of more unsaturated Ω‐9 acids. However, under conditions of severe Ω‐3 and Ω‐6 deprivation, humans engage in the elongation and desaturation of OA to generate mead acid (C20:3, Ω‐9) (Saini and Keum 2018; Innes and Calder 2018).

Ω‐6 and Ω‐9 fatty acids provide unique physiological benefits. Ω‐6, exemplified by LA, is crucial for cell membrane stability and plays a role in the inflammatory response. GLA, an Ω‐6 fatty acid, shows promise in promoting skin health and neurological development. On the other hand, Ω‐9, notably OAs, contributes to cardiovascular health by improving lipid profiles and exhibits anti‐inflammatory effects. OA positively influences insulin sensitivity and stabilizes cellular membranes, impacting cellular communication (Saini and Keum 2018; Innes and Calder 2018).

## 2 Sources of Omega Fatty Acids

*Saliva hispanica* L., commonly known as CS, is an excellent source of Ω‐3 essential fatty acids. This fatty acid profile is fully used in the physiological function of the skin; for example, it inhibits water loss and maintains the outermost layer of skin, the mature epidermis, as an effective barrier. For 5500 years, CS has been used as a food ingredient and for nutritional purposes in Mexico, and in recent times, it is used as a food ingredient in many countries (de Falco et al. 2017; Kobus‐Cisowska et al. 2019; Khalid et al. 2023). CS mainly presents PUFAs, which are essential for human and animal health (Diwakar et al. 2014). CS contains essential omega fatty acids such as OA and ALA. It is widely used in food applications, depending on its nutritional characteristics, as well as treating nervous system disorders. In the first step, an extract of CS was used to assess its ability to act as enzyme inhibitors (bacterial cholinesterase and acetylcholinesterase). Color extracts were found to be very effective against acetylcholinesterase and bacterial cholinesterase. In addition, an increase in the activity of the extract was shown in the grain filtration process (Rajput et al. 2021).

**FIGURE 1:**
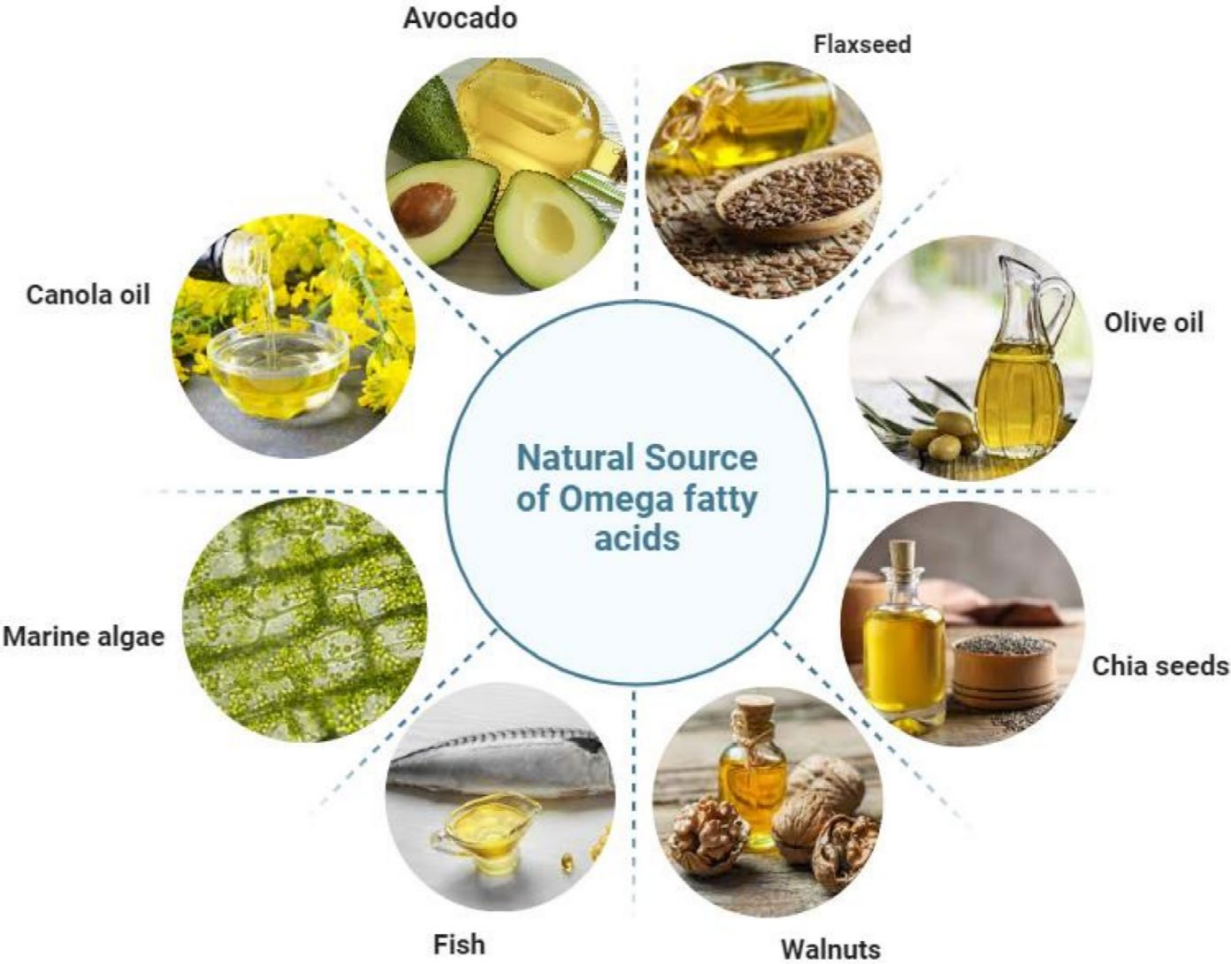
Natural sources of omega fatty acids.

Ashaf‐Ud‐Doulah et al. (2019) conducted research work on the chemical analysis of three common farm fish species, namely mrigal carp, rohu, and South Asian carp, to determine the fatty acid profile. These fish species emerged as a commendable source of PUFAs and Ω‐3, offering a combination of lean protein. Notably, the study revealed that the protein content in the fish ranged from 20 to 23 g/100 g, aligning closely with the protein content of the experimental feed, which measured at 67.70 g/100 g. Furthermore, the lipid content in the fish fillets fell within the range of 2.57–3.11 g/100 g, emphasizing a relatively low‐fat composition. In contrast, the feed exhibited a higher lipid content at 14.09 g/100 g. These findings underscore the nutritional value of the fish species, emphasizing its richness in protein and favorable fatty acid profile, particularly Ω‐3, making it a desirable component for a balanced diet. Additionally, the Ω‐3 and Ω‐6 series of PUFAs were computed across all fish species within the range of 1.69%–1.91%. Many sources of long‐chain PUFAs have been used to improve health benefits (Figure 1). FO is most commonly used for pharmaceutical purposes and supplements. This tropical FO is used as an experimental food product because it has been described as an excellent source of essential fatty acids, and health agencies have recommended these fatty acids as food supplements for children (Njinkoue et al. 2016).

FO is found to be an excellent source of LCPUFAs. The use of FO in food supplements is limited due to oxidative stability. Solutions to prevent this effect are described in detail in further parts of this review article. Another research work provided by Lawlor et al. (2010) studied the effect of a FO derived as a source of Ω‐3 series of PUFAs in the form of encapsulation. These microcapsules were used in the feed of the white lignin‐laying hens. Ninety‐six laying hens were used, and the groups were divided into four treatments (Peinado et al. 2016; Castejón and Señoráns 2020).

Flaxseed oil has been recognized as an excellent source of PUFAs in the Ω‐3 series, which is used for food supplementation and nutrition. Flaxseeds are used as food sources and have many valuable nutritional properties. Flaxseed oil is used to produce fibers. Flaxseed oil is mainly attributed to biologically active ingredients due to its nutritional benefits in humans, such as dietary lignan, unusual proteins, and ALA. It is reported that flaxseed oil is the richest natural source of Ω‐3 and ALA (Mahmudiono et al. 2022; Shim et al. 2024). Notably, flaxseed oil exhibits a composition low in saturated fatty acids (9%), moderate in monounsaturated fatty acids (18%), and abundant in PUFAs (73%) (Tonon et al. 2011). The predominant fatty acid in flaxseed oil is ALA, ranging from 39.0% to 60.4%, followed by oleic, linoleic, palmitic, and stearic acids, contributing to an excellent Ω‐6–Ω‐3 fatty acid ratio of approximately 0.3:1 (Sun et al. 2021). Despite its natural richness in antioxidants like tocopherols and β‐carotene, traditional flaxseed oil is susceptible to oxidation after extraction and purification (Saleem et al. 2024). Additionally, the bioavailability of ALA is influenced by the form of flax consumed, with ALA exhibiting greater bioavailability in oil than in milled seed and greater bioavailability in oil and milled seed than in whole seed (Austria et al. 2008).

Walnuts constitute a substantial reservoir of proteins, minerals (such as calcium, magnesium, phosphorus, and potassium), vitamins (including niacin, tocopherols, vitamin C, vitamin B6, and folate), fatty acids, and an array of phytochemicals encompassing phenolic compounds, phytosterols, and tocopherols (Usda 2019). Walnut consumption is linked to a variety of health benefits, notably the reduction of serum LDL and total cholesterol levels, imparting cardiovascular advantages (Bashan and Bakman 2018). The walnut kernel is reported to comprise 40%–70% oil, contingent on the specific variety. The composition of walnut oil encompasses diverse fatty acids, manifesting in saturated fatty acids, monounsaturated fatty acids, and PUFAs. In contrast to the prevalence of monounsaturated fatty acids in most nut oils, walnut oils exhibit a richness in PUFAs (Song et al. 2022). Within walnut oil, unsaturated fatty acids (monounsaturated fatty acids and PUFAs) include palmitoleic (16:1), oleic (18:1), linoleic (18:2), ALA (18:3), 11‐eicosenoic (20:1), and eicosapentaenoic (20:5) acids, constituting over 86% of the total fatty acids. Oleic and LA emerge as the most abundant monounsaturated fatty acids and PUFAs, respectively (Tindall et al. 2019; Hama and Fitzsimmons‐Thoss 2022). Approximately 7%–14% of the total fatty acids in walnut oil comprise saturated fatty acids, including myristic (C14:0), palmitic (C16:0), stearic (C18:0), arachidic (C20:0), and behenic (C22:0) acids, with palmitic acid notably surpassing other saturated fatty acids. Walnut oil exhibited a higher percentage of saturated fatty acids than heartnut oil, while its PUFAs content was lower (Gao et al. 2019; Nguyen and Vu 2023).

Canola oil is characterized by a composition comprising 6%–14% of ALA, 50%–66% OA, and a minimal quantity (< 7%) of saturated fatty acids. The presence of ALA in canola oil is of particular significance, as it is an essential fatty acid associated with various health benefits (Bauer et al. 2015). These benefits include inhibiting eicosanoid production, modulation in the synthesis of several prostanoids, reduction of blood pressure, mitigation of serum triglyceride and cholesterol levels, and impeding the growth of tumors. The predominant fatty acids present in the oil are oleic (56.8%–64.9%), linoleic (17.1%–20.9%), and palmitic (4.18%–5.01%) acids (Ghazani and Marangoni 2013). Various tocopherols were identified in both rape and canola oils in varying proportions, including α‐tocopherol, γ‐tocopherol, δ‐tocopherol, β‐tocopherol, and α‐tocotrienol. Among these, the primary tocopherols in the seed oil of canola cultivars were α‐tocopherol (13.2%–40.0%) and γ‐tocopherol (33.6%–51.5%), accompanied by α‐T3 (0.0%–1.34%) and δ‐tocopherol (0.25%–1.86%) (Matthaus et al. 2016).

Olive oil ( *Olea europaea* L.) stands out as a prominent reservoir of monounsaturated fatty acids, specifically OA, categorized as an Ω‐9 fatty acid. The lipid profile of olive oil exhibits a noteworthy concentration of OA, ranging from 55% to 83%, contingent upon varietal distinctions and processing methodologies. The Ω‐9 fatty acids found in olive oil have been implicated in cardiovascular health by virtue of their involvement in the regulation of lipid metabolism, modulation of inflammatory processes, and potential influence on insulin sensitivity (Medeiros‐de‐Moraes et al. 2018). Consumption of olive oil, abundant in Ω‐9 fatty acids, has been associated with diverse health advantages, including enhancements in lipid profiles and a diminished susceptibility to cardiovascular diseases. Furthermore, the combination of antioxidants and bioactive compounds inherent to olive oil contributes substantively to its nutritional appeal, rendering it a valuable dietary constituent with implications for human health (Gorzynik‐Debicka et al. 2018).

Marine microalgae stand out as a significant source of Ω‐3 fatty acids, specifically EPA and DHA. These PUFAs play pivotal roles in cellular membrane composition and contribute essential functions to cardiovascular and neurological systems (Santigosa et al. 2020). The intrinsic capability for de novo biosynthesis of EPA and DHA is inherent in select strains of marine microalgae. Despite conventional associations of Ω‐3 fatty acids with marine fauna, recent attention has centered on the algal kingdom as a primary source. This paradigm shift aligns with environmental sustainability objectives, circumventing concerns tied to overfishing and concurrently offers a viable, plant‐derived alternative suitable for individuals embracing vegetarian or vegan dietary regimens. The elucidation of marine algae's role as an immediate and eco‐friendly reservoir of indispensable omega fatty acids underscores its scientific significance in addressing human nutritional needs (Martins et al. 2013; Moomaw et al. 2017). Attempting to understand the genomes and transcriptomes of several high‐value algal species, as well as their metabolic pathways leading to the biosynthesis of carotenoids, lipids, and PUFAs, has advanced recently (Leu and Boussiba 2014; Rastogi et al. 2018; Fu et al. 2019). Recent advancements in genetic engineering and microalgae transformation have made it feasible to use metabolic engineering to boost the production efficiencies of bulk biomass, biofuels, and high‐value products in microalgae (Moomaw et al. 2017). In animal‐based sources of omega fatty acids, the small and fatty mackerel (fish) is frequently consumed as whole fillets and smoked. It offers 130% of the daily value (DV) for selenium and 500% of the DV for vitamin B12. Ω‐3 composition: A serving of 3.5 oz (100 g) contains 4580 mg of EPA and DHA combined. Likewise, salmon is a nutrient‐rich food that contains B vitamins, vitamin D, selenium, and high‐quality protein. Regular salmon consumption is linked to a decreased risk of depression, dementia, and heart disease. Ω‐3 content: 3.5 oz (100 g) servings contain 2150 mg of EPA and DHA (combined). Moreover, a supplement made from the livers of cod fish is called cod liver oil. It is high in vitamins A, D, and Ω‐3. Ω‐3 content: each tablespoon contains 2438 mg of EPA and DHA combined. Additionally, a medium‐sized oily fish, herring is frequently pickled or cold‐smoked. A serving of 3.5 oz (100 g) contains 779% of the DV for vitamin B12 and nearly a hundred percent of the DV for selenium. Ω‐3 content: 3.5 oz (100 g) servings contain nearly 2150 mg of EPA plus DHA (mixed). One of the healthiest foods and a fantastic source of Ω‐3 fatty acids is oysters, which are high in zinc. 329 mg of Ω‐3 per serving were reported by Ponnampalam et al. (2021).

Talking about some drawbacks of omega fatty acids, based on food or feed categories, one common food derived from animals is red meat, which includes beef, lamb, and pork. Long‐chain Ω‐3 fatty acids (EPA and DHA) are not abundant in red meat, despite the fact that it contains vital nutrients. Ω‐3 is generally found in lower concentrations in the tissues of animals raised on grain‐based diets, which are typical in feedlots. When consumed in excess, red meat's Ω‐6–Ω‐3 fatty acid ratio can be detrimental to one's health (Lakra et al. 2019). Because of their natural diet, animals raised on grass have higher levels of Ω‐3 fatty acids. Animals raised on grain, which are frequently found in feedlots, on the other hand, have less Ω‐3 in their bodies. Selecting grass‐fed meat could result in improved Ω‐3 profiles. Although necessary, Ω‐6 fatty acids can be dangerous in excess. With a higher intake of Ω‐6, modern diets frequently contain an imbalance. When Ω‐6 is consumed more than Ω‐3, it can cause inflammation and other health problems. These fatty acids must be in a balanced ratio for optimum health (Saidaiah et al. 2024). Conclusively, the best source of EPA and DHA is still fish, particularly fatty fish such as sardines, mackerel, and salmon. Flaxseed, CS, and walnuts are examples of plant‐based sources of ALA, which are less effective at converting to EPA and DHA. Additionally, well‐liked are supplements (like FO and algae‐based).

## 3 Extraction Techniques

### 3.1 Traditional Techniques of Extraction

#### 3.1.1 Solvent Extraction

Solvent extraction was conducted using petroleum benzene (boiling point 40°C–60°C) and a mixture of methanol–water–petroleum benzene (90:5:5, v/v/v) with a 15% moisture content. Oil extraction from seeds was performed with a Soxhlet apparatus using varying ratios and extraction times for optimization (López‐Bascón and de Castro 2020).

FO was extracted from the swamp eel (head and fillet) using a solvent extraction method. The fat ratio in fish fillets was between 0.50 and 1.06 g·100 g^−1^ and in the head, 0.40–0.78 g·100 g^−1^. The major fatty acids in the FO are extracted from the head and body, such as DHA, arachidonic acid, hexadecanoic acid, and monounsaturated Ω‐9 fatty acid. Fatty acid (DHA and arachidonic acid) in body oil ranged from 8.25 to 6.21 g·100 g^−1^. The fatty acid contents in the head oil ranged from 6.11 to 8.77 g·100 g^−1^. The percentages of arachidonic acid and DHA content of adipocere body oil were 10.1% and 7.16%, respectively (Razak et al. 2001). FO was extracted from freshwater *Labeo catla* samples using the solvent extraction method at optimum conditions to determine the calorimetric properties and fatty acid profile. The results showed that the fatty acids analysis of FO contained total saturated fatty acids and total unsaturated fatty acids (55.5% and 41.1%). Furthermore, LCPUFAs (21.6%), DHA (7.63%), and monounsaturated fatty acids (19.5%) were measured (Andhale et al. 2017) (Table 1).

**TABLE 1:** Extraction methods of oilseeds and oils rich in omega fatty acids.

Birthplaces	Category traditional technologies‐A green technologies‐B	Extraction	Preservation methods and optimum conditions	Active components	Results	References
CS	A	Solvent extraction by binary water‐acetone mixture (1/3–2/3)	—	LA β‐tocopherol Antioxidant potential Ferric reducing ability	250.20 μmol TE/g; 720.15 μmol TE/g	Teh et al. (2015b); Harris et al. (2019)
Chia seed oil (CSO)	A	Solvent extraction Ternary mixture Water‐ethanol‐acetone	—	LA ϒ‐tocopherol	990.15 μmol TE/g 370.53 μmol TE/g 60.96 mg GAE/g	Alcântara et al. (2019)
Flaxseed oil	A	Solvent extraction method using two solvents (petroleum benzene and methanol–water‐petroleum benzene)	Preservation of Ω‐3 fatty acids was kept under a nitrogen atmosphere at −30°C up to 90 days storage	ALA, Ω‐3 and Ω‐6 fatty acids	Highest yield of linolenic acid, improved linseed oil oxidative stability	Hassan‐Zadeh et al. (2008)
CSO	B	Cold press extraction method	Heat treatment through microwave oven at 900 W	Rosmarinic acid Caffeic acid		Özcan et al. (2019)
FO	B	Supercritical fluid extraction method	Microencapsulation of fish oils through spray drying method using maltodextrin and cellulose as wall materials	—	Improved oxidative stability of FO powder The lowest value of conjugated linoleic acid (CLA) with lipid oxidation was obtained at aw (0.743) at 35°C for whey protein concentrate	Kuvendziev et al. (2018)
CSO	B	Supercritical fluid extraction method	Multilayered microencapsulation using maltodextrin and chitosan as wall material		Increased oxidative stability Positive 60 days storage period	Shahidi and Ambigaipalan (2018)
Hake offcuts oil	B	Supercritical CO_2_ extraction at 313 k temperature and 25 MPa pressure	Encapsulation of Ω‐3 PUFAs, to be then incorporated into different food products	Total Ω‐3 and Ω‐6 fatty acids in hake offcuts is 132 mg/g and 19 mg/g of FO.	Supercritical CO_2_ extraction prevents lipid oxidation and removes the amount of pollutants	Venugopalan et al. (2021)
Salmon offcuts oil	B	Solvent‐based extraction, with the combination of methanol and chloroform	Nanoparticle of Ω‐3 oils, converting them into powdered form	Total Ω‐3 fatty acids 100 mg/g and total Ω −6 fatty acids 108 mg/g of FO.	Best oil quality is obtained regarding lipid oxidation, unpollutants and improved sensory properties	Rubio‐Rodríguez et al. (2008)
Jumbo squid liver oil	B	Supercritical CO_2_ extraction at 313 k temperature and 25 MPa pressure	Spray drying converts the Ω‐3 PUFAs into powdered oil and colloidal dispersion forms	Ω‐3 PUFAs (284 mg/g of oil) Ω‐6 PUFAs (29 mg/g of oil)	Facilitates storage by extending the shelf life	Kris‐Etherton et al. (2002)
Orange rough offcuts oil	B	Supercritical fluid extraction	Encapsulation with biopolymers	Ω‐3 PUFAs (16 mg/g of oil) Ω‐6 PUFAs (8 mg/g of oil)	Safe and stable FO powder for bioactivity	Gaber et al. (2018)
FO	B	Supercritical fluid extraction	Nanoencapsulation and microencapsulation	DHA and EPA	Economical oil of high purity and yield, without solvents and high temperature	Rubio‐Rodríguez et al. (2008)
Canola oil	B	Cold press‐ super critical CO_2_ extraction	Oxidative stability because of a high amount of antioxidants (tocopherols, phytosterols and canolol)	Linolenic acid, OA, palmitic acid, lauric acid.	Enhance oil yield and quality	Gaber et al. (2018)
Avocado oil	B	MAE with ethanol	The electric field (9kVcm‐1720 Hz, 5‐25 min) method is used as a preservative alternative to antioxidants to reduce oxidative stability	Palmitic acid, Estearic acid, palmitoleic, oleic, linoleic and ALA.	Inactivated polyphenol oxidase enzymes thus help in the conservation of oil	Flores et al. (2019)
Walnut oil	B	Cold press extraction method	Stored in a dark place at ambient temperature	LA, linolenic acid and Ω‐3 PUFAs	Shelf life more than 4 months	Masoodi et al. (2022)

Abbreviations: Ω‐3, Omega‐3; Ω‐6, Omega‐6; ALA, alpha‐linolenic acid; CLA, conjugated linoleic acid; CS, chia seeds; CSO, chia seed oil; DHA, docosahexaenoic acid; EPA, eicosapentaenoic acid; FO, fish oil; LA, linoleic acid; MAE, microwave‐assisted extraction; PUFAs, polyunsaturated fatty acids.

### 3.2 Green Technologies

The utilization of organic solvents for the isolation of oils shows drawbacks: (i) elevated temperatures lead to the degradation of heat‐sensitive Ω‐3 PUFAs, (ii) the potential retention of toxic residues from organic solvents in the final product, and (iii) the environmental ramifications associated with organic solvent usage (Pinheiro Pires et al. 2019). Consequently, alternative approaches rooted in green chemistry are being developed to address these concerns, embracing methods such as supercritical fluid extraction, enzyme‐assisted extraction, microwave‐assisted extraction (MAE), and ultrasound‐assisted extraction (UAE). Supercritical fluid extraction (SFE) is the most extensively employed green method for extracting Ω‐3 marine oils. This is attributed to its capacity to economically produce oils characterized by high purity and yield, all achieved without requiring elevated temperatures or organic solvents (Senanayake 2013; Rahim et al. 2022).

#### 3.2.1 Microwave‐assisted extraction (MAE)

Microwave pretreatment of oilseeds offers a compelling alternative to traditional extraction methodologies. Scientific investigations have established that MAE enhances the overall oil yield and positively influences the phytochemistry and functional attributes of the resulting oil compared to oils obtained through conventional means. Notably, MAE reduces both the extraction duration and solvent volume requirements (Castro‐Muñoz et al. 2024; Hernández‐Pinto et al. 2024).

Furthermore, oils extracted via microwave roasting exhibit heightened oxidative stability, extending shelf life. Additionally, this technique augments the extracted oils' antioxidant capacity and phenolic profile. In MAE, the specimen undergoes exposure to non‐ionizing radiation within the frequency range of 300 MHz–300 GHz (de la Fuente et al. 2022). These electromagnetic radiations induce ionic conduction, compelling molecular alignment with the electric field of radiation. This phenomenon generates heat, leading to the evaporation of water content within the specimen and an increase in pressure within the cellular structure. Consequently, tissue cells experience rupture under elevated pressure, facilitating the release of bioactive constituents for extraction. MAE is also denoted as solvent‐free microwave extraction due to the absence of solvents. The method's efficacy depends on microwave frequency, extraction duration, and initial water content within the sample matrix (Pinela et al. 2022). MAE is a method used for extracting oil from by‐products of fish processing. Although not widely adopted on its own, MAE is typically combined with traditional extraction techniques to enhance oil yield and reduce lipid oxidation (Ivanovs and Blumberga 2017). Chimsook and Wannalangka (2015) reported that employing MAE at 110 W for 60 s before enzymatic hydrolysis with alcalase improved oil extraction from catfish waste. Similarly, Bruno et al. (2019) observed positive results using MAE as a pre‐treatment for oil extraction from rohu fish heads.

#### 3.2.2 Pressurized Liquid Extraction (PLE)

Alternatively recognized as accelerated solvent extraction (ASE), PLE represents a burgeoning extraction methodology, which employs a blend of water and solvent under elevated temperatures and pressures to extract bioactive constituents (Ferreyra‐Suarez et al. 2024).

Oils obtained through PLE exhibit heightened concentrations of phenolic acids, polymethoxylated flavones, terpenoids, and polyphenols, among other compounds. Due to their rich phytochemical profile, the resultant extracts manifest elevated antioxidant and radical scavenging activities (Villanueva et al. 2019). The application of high pressure enables liquids to attain temperatures surpassing their atmospheric boiling points without reaching the boiling state. This configuration amplifies the solubility and diffusion of bioactive components into the solvent. Concurrently, it markedly diminishes the surface tension and viscosity of the solvent. Collectively, these factors facilitate the optimal extraction of target components after the thorough drainage of the sample matrix. PLE is executed in a dynamic setup, wherein the solvent is delivered at a constant flow rate. Alternatively, the static mode of operation involves solvent replacement in one or more cycles within a predetermined timeframe (Alvarez‐Rivera et al. 2020).

#### 3.2.3 Cold‐Pressed Extraction

Cold‐pressed extraction is conducted at low temperatures, obviating the need for solvents or additional sample processing. Extracts derived from cold pressing exhibit a concentrated phytochemical profile attributable to the low‐temperature conditions and the absence of solvents or chemical agents throughout the process (Mihai et al. 2020). Furthermore, oils extracted via this technique demonstrate elevated levels of total tocopherol, oxidative stability, and antioxidant properties compared to solvent extraction. Similarly, this method ensures the attainment of oils with high quality and concentration of bioactive compounds. While cold‐press extraction is recognized for its cost‐effectiveness, environmental friendliness, and safety (Chandra et al. 2020).

#### 3.2.4 Ultrasound‐assisted extraction (UAE)

UAE has gained widespread preference as an extraction method owing to its advantages, including low operating temperature, minimal solvent requirements, and shortened processing time. Scientific investigations have determined that oils extracted using UAE exhibit negligible structural and functional alterations in fatty acids (Mushtaq et al. 2020). Furthermore, such oils demonstrate elevated concentrations of polyunsaturated and monounsaturated fatty acids and reduced levels of saturated fatty acids. The abbreviated extraction duration contributes to enhanced oil stability, and evidence suggests that the UAE may augment the antimicrobial properties of the extracted oils (de Mello et al. 2017). Ultrasonication parameters typically range from tens to hundreds of watts, with frequencies usually falling within the 20–40 kHz range and varying time durations. Regarded as a green technology, ultrasound offers advantages such as lower costs, reduced operational expenses, higher yields, and decreased energy consumption. The efficiency of ultrasound arises from the disruption of cell walls and mass transfer facilitated by cavitation bubbles generated by ultrasound radiation, consequently promoting the mass transfer of phytochemicals. Bioactive compounds are released through processes such as diffusion or dissolution (Ivanovs and Blumberga 2017).

#### 3.2.5 Supercritical Fluid Extraction

Supercritical fluid extraction revolves around utilizing a supercritical fluid as its primary component. A substance's triple point represents the conditions under which it coexists in all three phases: solid, liquid, and gas at a specific temperature and pressure. As temperature and pressure increase, the phase differences between solid, liquid, and gas diminish, culminating in the critical point (Yousefi et al. 2019). At this critical point, the substance exhibits a liquid's solubility and density characteristics while behaving like a gas. For carbon dioxide (CO_2_), the critical point is defined by a critical temperature of 31°C and a critical pressure of 74 bar. Extraction in this method occurs through two distinct stages: diffusion and dissolution. The supercritical CO_2_ or propane (as a substitute for the hexane extraction of oil), behaving like a gas, readily permeates the solid matrix and can penetrate small pores within the sample (Ahmad et al. 2019). The high solubility and density of supercritical CO_2_ facilitate the extensive dissolution of the extract, transporting it from the solid matrix to the outer layer and subsequently into the solution. CO_2_ is a preferred solvent in this technique due to its widespread availability in the environment, recyclability, absence of residue within the sample, and low critical temperature and pressure (Rahim et al. 2023).

**FIGURE 2:**
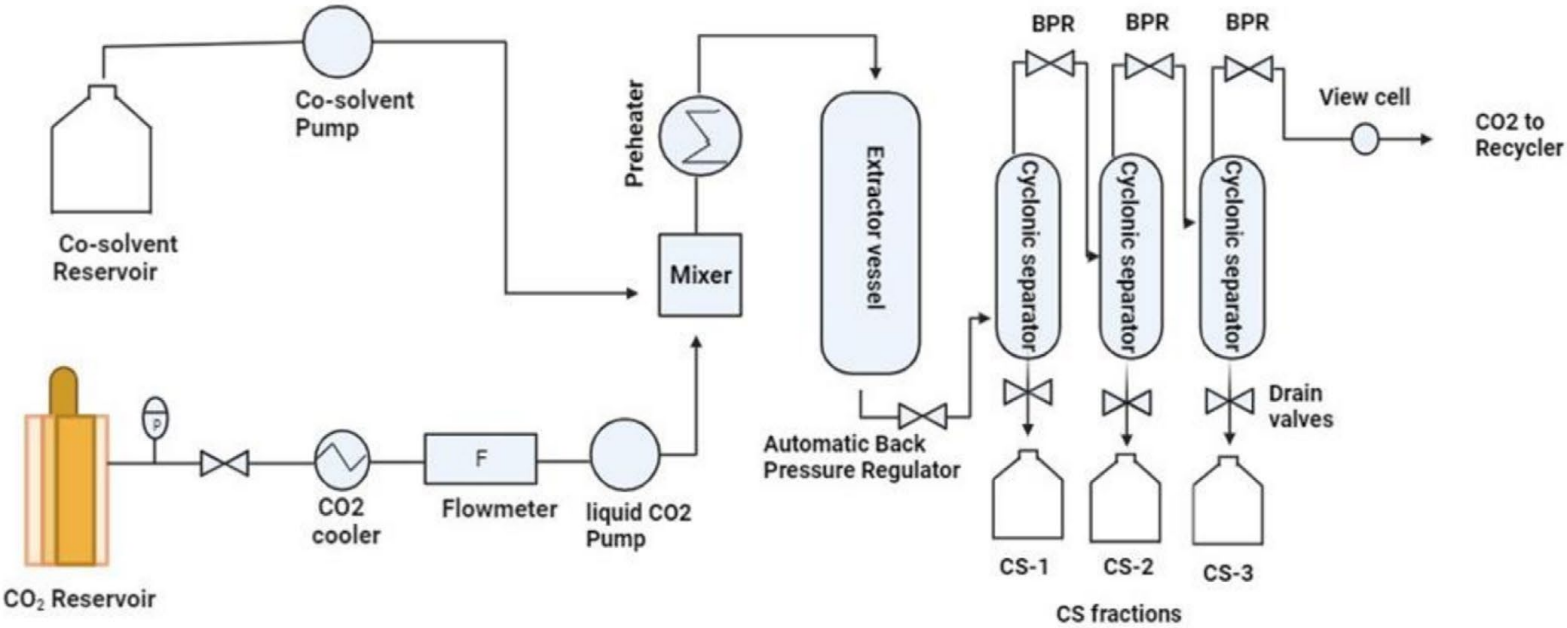
Diagrammatic representation of the process flow for supercritical CO_2_ extraction.

CO_2_ is the most widely employed solvent in its supercritical state due to its relatively low critical temperature and pressure, standing at 30°C and 7 MPa, respectively. Additionally, CO_2_ is characterized as non‐toxic, non‐corrosive, non‐flammable, inert, and cost‐effective. Furthermore, the solvent can be easily decompressed post‐extraction, and CO_2_ can be separated or re‐condensed for subsequent reuse. Consequently, the oil and extracted meal can be obtained without necessitating further solvent removal treatments. As represented in Figure 2, the scheme explains a proposed supercritical extraction process for oil extraction (Aytac 2022; Machmudah et al. 2024).

The enhancement of oil shelf life is contingent upon oxidative stability, a pivotal factor in this regard. Augmenting the quantity of antioxidant components, including tocopherols, canolol, and phytosterols, heightens the oil's oxidative stability. In the present study, the impact of utilizing supercritical CO_2_ as a solvent on the oxidative stability of canola oil is investigated, compared to that of *n*‐hexane solvents. The findings indicate a positive influence of CO_2_ on oxidative stability, attributable to an escalation in the concentration of antioxidant components. Notably, the phytosterol content experienced a 51% increase compared to canola oil extracted using *n*‐hexane (Przybylski et al. 1998; Ahangari et al. 2021).

#### 3.2.6 Enzyme‐Assisted Extraction

The enzyme‐assisted extraction method employs specific enzymes to facilitate the extraction of bioactive compounds. This method is environmentally friendly and favored for avoiding denaturing conditions, such as solvents, high temperatures, and light. Its advantages encompass catalytic efficiency, mild aqueous conditions, high specificity, biological activities, high yields, low energy consumption, and cost‐effectiveness. Commonly utilized enzymes in this method include pectinases, amylases, proteases, and cellulases. Research indicates that extracts obtained using pectinase enzymes exhibit enhanced immunostimulatory activities (Liu et al. 2016). Furthermore, it has been reported that extracts obtained through enzyme‐assisted extraction possess more than double the antioxidant activity compared to extracts obtained through conventional methods. This technique involves the catalytic metabolism of polymers in the cell wall, such as hemicellulose and cellulose. Enzymes facilitate the breakdown of cell walls, releasing bioactive compounds that may be bonded to the cell wall through hydrogen bonding or enclosed within hydrophobic pockets. Consequently, these enzymes liberate bioactive components, facilitating their subsequent extraction. Moreover, the high specificity of the enzymes employed in the extraction process enables the development of tailored extraction formulations (Aitta et al. 2021).

#### 3.2.7 Pulsed Electric Field‐Assisted Extraction (PEF)

PEF extraction represents a non‐thermal methodology employing electric fields to extract bioactive compounds. Electric fields play a dual role, either enhancing the solubility of phytochemicals or augmenting the permeability of the cell membrane, thereby improving the extraction process. This technique facilitates the increased release of polysaccharides and proteins from the sample matrix while concurrently enriching the extract with phenolics, carotenoids, and other pigments (Burnett et al. 2022). Pulsed electric field application also heightens the oil's antioxidant properties, consequently enhancing the stability and extending the shelf‐life of the resulting product. The PEF technique is conducted in both batch and continuous systems, with electric field strengths ranging from 100 to 300 V/cm in batch systems and 20 to 80 V/cm in continuous systems, respectively (Teh et al. 2015a). The electric field strength and application duration depend on the cell type and extract. Two proposed mechanisms underline the effectiveness of this technique: first, the electric field induces structural changes in bioactive compounds, thereby increasing their solubility; second, the electric field heightens cellular permeability by creating hydrophilic pores in the cell membrane, leading to the release of phytochemicals. The nature of structural change, whether temporary or permanent, is contingent upon the energy of the applied electric field (Teh et al. 2015a).

## 4 Characterization

Assessing oils' nutrition, safety, and physicochemical attributes is contingent upon their chemical composition, emphasizing the need for suitable analytical methods to ascertain such composition. Gas chromatography (GC), frequently coupled with mass spectrometry (MS), is extensively used to analyze oil samples' non‐polar lipids. In this process, the oils undergo chemical treatment, converting triacylglycerols into volatile free fatty acid methyl esters (FAMEs), which can then be separated utilizing appropriate GC columns (Teh et al. 2015b). The resulting chromatogram exhibits a series of peaks corresponding to fatty acids with diverse chain lengths and degrees of unsaturation. Traditional quantification involves measuring peak areas and identifying fatty acids by comparison with established standards. Enhanced analysis can be achieved by coupling the GC instrument with an MS (Waktola et al. 2020).

The investigation and identification of diverse FAMEs which are renowned as Ω‐3 fatty acids having carbon chain lengths of C20 and C22, exemplified by arachidonic acid (20:1), EPA (20:5), and DHA (22:6), along with their positional and geometrical isomers, has been extensively explored through the application of GC and Gas Chromatography–Mass Spectrometry (GC–MS) techniques across various sample types (Karasek and Clement 2012; Lovestead and Urness 2019). Generally, the GC analysis of FAMEs involves multiple steps, including fatty acid esterification, injection, separation, identification, and quantification. State‐of‐the‐art fused silica capillary columns, commercially available today, exhibit exceptional capabilities in separating FAMEs, even in complex biological samples (Moeder 2014; Guo 2014). Specialized polar stationary phases, such as Sil‐88 (comprising 100% cyano‐propylpolysiloxane), are employed for high‐resolution FAMEs analysis. However, these columns have a drawback of limited thermal stability, resulting in prolonged retention times, restricting their maximum operating temperature to 500 K. Conversely, non‐polar stationary phases possess superior thermal stability, a broad range of operating temperatures, and chemical inertness, albeit with compromised resolution. Leveraging these advantages, non‐polar phases prove effective in analyzing fatty acids with higher molecular masses (Sahil et al. 2011).

Furthermore, advanced high‐resolution nuclear magnetic resonance (NMR) methods offer crucial insights into the types of lipids present in fish oils. Thin‐layer chromatography analysis provides additional information about lipid types, including triacylglycerols, diacylglycerols, monoacylglycerols, free fatty acids, and phospholipids. These analytical methodologies are integral to the burgeoning field of lipidomics, which aims to elucidate the structure and function of diverse lipid molecules within biological samples (Li et al. 2017). The extracted oils' extraction yield and Ω‐3 content were quantified using gas chromatography. FAMEs were prepared following the AOCS standard method. GC analysis utilized a fused‐silica capillary column with 60 m × 0.25 mm × 0.39 μm film thickness dimensions. A split injector (1 μL injection) operated at 240°C, and a flame ionization detector (FID) at 250°C were employed. Helium served as carrier gas at a pressure of 50 psi, while the column temperature was maintained at 190°C (Eibler et al. 2017; Rahim et al. 2024).

## 5 Oxidation of Omega Fatty Acid

Oxidation is the most common factor affecting the stability of long‐chain PUFAs at room temperature. Omega oxidation is a metabolic pathway that serves as an alternative to beta‐oxidation for the catabolism of fatty acids. This pathway is important when beta‐oxidation is impaired or when some fatty acids are unsuitable substrates for beta‐oxidation. Omega oxidation occurs in the smooth endoplasmic reticulum of liver and kidney cells, where it initiates the oxidation process at the omega carbon, the terminal carbon atom farthest from the carboxyl group of the fatty acid (Arnold et al. 2010). The process begins with the hydroxylation of the omega carbon, catalyzed by cytochrome P450 enzymes, resulting in the formation of a hydroxyl group. Subsequently, this hydroxyl group undergoes dehydrogenation to form an aldehyde, which is further oxidized to yield a dicarboxylic acid. These dicarboxylic acids can then enter the mitochondria, where they undergo beta‐oxidation, ultimately producing Acetyl‐CoA units that feed into the citric acid cycle for energy production (Van Bogaert et al. 2011). The omega oxidation pathway plays a crucial role in maintaining metabolic flexibility, especially under conditions where beta‐oxidation is compromised. For instance, in certain metabolic disorders where beta‐oxidation is defective, omega oxidation becomes a vital compensatory mechanism to prevent the accumulation of unoxidized fatty acids (Edson and Rettie 2013). Additionally, omega oxidation contributes to the detoxification of xenobiotics and the metabolism of specific fatty acids that are poor substrates for beta‐oxidation due to structural constraints, such as the presence of methyl branches or double bonds at particular positions. By converting these fatty acids into more water‐soluble dicarboxylic acids, omega oxidation facilitates their excretion or further metabolism, thereby maintaining lipid homeostasis and preventing lipotoxicity. Therefore, the peroxide value of omega‐enriched oil decreases during the storage period (Miura 2013). Because PUFAs oxidize when exposed to air, high temperatures, and moisture, adding them to food products can cause many problems. Unwanted flavors and a reduction in nutritional value are the outcomes of this oxidative deterioration. To increase the stability of edible oils high in PUFAs against oxidation, a variety of techniques have been used, such as physical blending, isomerization, and encapsulation (Homroy et al. 2024).

### 5.1 Solubility Issues

Elevated plasma fatty acid levels are observed in various metabolic disorders. The detrimental cellular effects of fatty acid excess, which lead to lipotoxicity, are frequently investigated in vitro to gain insights into the mechanisms involved in these conditions. Since fatty acids have low solubility, they are typically studied when bound to proteins like bovine serum albumin (BSA). Preparing the solutions for the conjugation of fatty acids to albumin necessitates attention to several aspects, such as the effective concentrations of free fatty acids, the selection of different fatty acid types, variations in BSA, suitable controls, and ensuring that cells effectively take up the fatty acids (Alsabeeh et al. 2018).

### 5.2 Physiochemical Stability

The physiological stability and bioavailability are chief alarms in the fortification of DHA and EPA. The omega fatty acid nanoemulsions, using plants (sunflower) as oil carriers, are industrialized as impulsive and low‐slung energy systems (Inapurapu et al. 2020). However, food‐derived bioactive compounds are also reported to be hampered by different physicochemical and physiological barriers that limit their oral bioavailability (Gleeson et al. 2016).

### 5.3 Preservation Technologies

Integrating health‐promoting Ω‐3 PUFAs into supplements, pharmaceuticals, and functional foods faces constraints. These unsaturated fatty acids are vulnerable to oxidative degradation, creating the potential for developing unfavorable aromas, commonly called “rancidity,” thereby diminishing consumer acceptance (Julio et al. 2019). Furthermore, certain by‐products resulting from lipid oxidation reactions demonstrate toxicity, posing a risk of inducing chronic health issues when regularly consumed over extended durations (Zhuang et al. 2018). A formidable obstacle in developing Ω‐3‐enriched food and beverage products lies in the markedly low solubility of fish oils in water. Furthermore, the bioavailability of Ω‐3 PUFAs can exhibit considerable variability, particularly contingent upon the delivery form. Bulk forms have demonstrated slower and less extensive absorption than emulsified forms (Timilsena et al. 2018).

Advanced encapsulation technologies present a viable solution to address these challenges. These methodologies involve the conversion of Ω‐3 PUFAs into colloidal structures, such as liposomes, lipid droplets, or biopolymer particles, which are subsequently incorporated into food or beverage matrices (Us‐Medina et al. 2018). These colloidal materials can be transformed into a powdered state in specific applications, facilitating handling, storage, utilization, and enhancing oxidation resistance. Various processing operations, including spray drying, freeze drying, and fluidized bed drying, can be employed (Pourashouri et al. 2014; de Melo Ramos et al. 2021). The judicious selection of an appropriate encapsulation technology can substantially enhance the chemical stability, water‐dispersibility, and bioavailability of Ω‐3 oils (Xiong, Zhang, et al. 2021).

The study explored the antioxidant properties and phenolic compounds in a binary mixture of water‐acetone and a ternary mixture, revealing significant scavenging capacity and reducing abilities. Microwave heating of CSO at various power levels induced changes in LA levels, PUFAs, tocopherols, and specific acids. The study also investigated water‐in‐oil and oil‐in‐water emulsions, demonstrating morphological changes and viscoelastic properties influenced by the emulsifier. Furthermore, efforts were made to enhance the peroxide value of CSO through microencapsulation, utilizing chia protein isolate gum and complex organic‐rich droplets as wall materials. Overall, the research highlighted the dynamic responses of CSO to different treatments and formulations, providing valuable insights into its chemical transformations and potential applications in food and health contexts (Yousefi et al. 2019; Khalid et al. 2023). The wall material properties, core and wall ratio, and the encapsulation and oxidative stability drying method were examined. Oxidative stability of microcapsules produced using chia protein isolate gum with a complex organic‐rich droplet as core material shows more efficiency when compared to the microcapsules generated using the wall material (chia gum and isolate). CS gum microcapsules showed low efficiency (67.3%) and oxidative stability (6.6 h). Furthermore, the chia protein isolate microcapsules revealed a higher efficiency of 93.9% and oxidative stability (12.3 h). CS gum, chia protein isolate, and complex organic droplets were used as wall material to produce microcapsules of CSO. Furthermore, the ratio between core and wall material in these microcapsules was shown to be 1:2, and storage stability was six times higher when compared with non‐capsulated CSO. The peroxide value was less than 10 mg/kg during 1 month of storage (Us‐Medina et al. 2018).

## 6 Encapsulation Technologies

Adapting oral delivery approaches using excipients from the pharmaceutical industry to nutraceuticals can enhance delivery. A tried‐and‐true method for improving the oxidative stability and functional characteristics of oils high in Ω‐3 fatty acids is encapsulation. To enhance and stabilize the delivery of Ω‐3 fatty acids in food products, a variety of encapsulation techniques have been developed. The intended use of the encapsulated oil determines which encapsulation technique is best. Furthermore, by encouraging greater uptake of the encapsulated form in the intestinal epithelium, encapsulation improves the bioavailability of Ω‐3 fatty acids (Homroy et al. 2024).

Clinical trials were performed to check the bioavailability of Ω‐3 fatty acids in humans. In these trials, Ω‐3 PUFAs supplementation has indicated health benefits in older adults (≥ 65) by mitigating age‐relevant muscle mass, neuromuscular, and physical weakness in patients (Dam et al. 2025). The mentioned meta‐analysis indicated that resistance training plus Ω‐3 PUFAs had a potential effect on muscle mass performance. Moreover, bioavailability can vary due to a change in the source dose intake. There are methodological limitations to generalize the standard protocols. Chronic studies amended with suitable biomarkers are needed to be addressed to access the clinical relevance of Ω‐3 PUFAs supplementation (Alijani et al. 2025).

**FIGURE 3:**
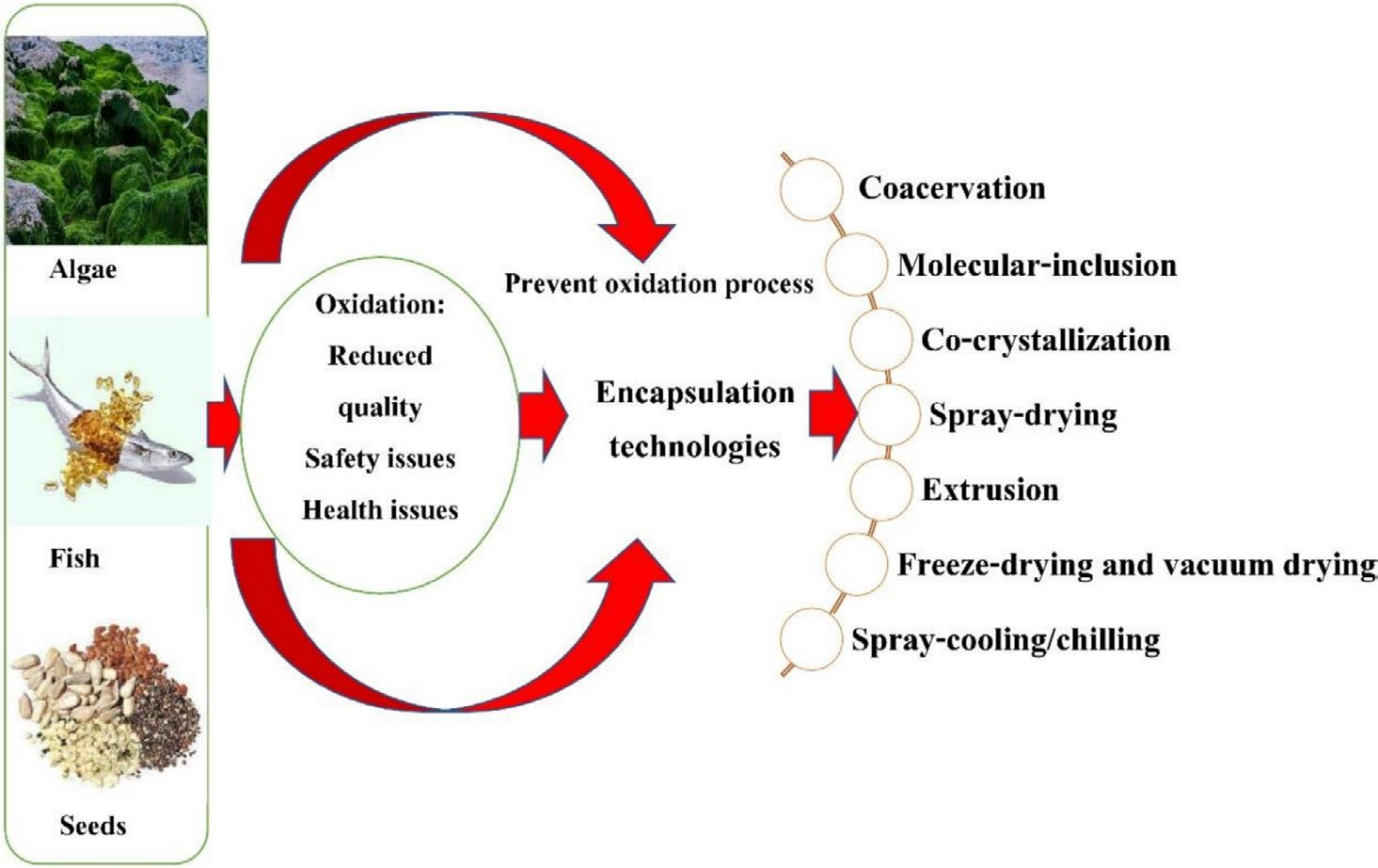
Preventing oxidation process of omega fatty acids.

A recent review study reported by Yavari et al. (2025) has mentioned that the impact of bioactive substances, such as Ω‐3 PUFAs and polyphenols, has demonstrated encouraging outcomes in different clinical trials for lowering amyloid‐β levels, enhancing cognition, and modifying the signaling pathways linked to Alzheimer's disease. The relationship between obesity, insulin resistance, inflammation, and Alzheimer's disease pathogenesis is examined in this study, with a focus on the potential of dietary components and their function in lowering oxidative stress, inflammation, and cognitive decline as effective preventative and therapeutic measures for Alzheimer's disease. Preventing the oxidation process of omega fatty acids is described in Figure 3.

### 6.1 Liposomes

Liposomes typically consist of phospholipids arranged in bilayer structures (Nsairat et al. 2022). They commonly consist of one or more bilayers organized into concentric rings, thus incorporating both non‐polar and polar regions, enabling the encapsulation of hydrophilic and hydrophobic substances. Various preparation methods, including solvent evaporation, injection, and micro‐fluidization methods, are available for liposome assembly, differing in commercial potential (Amoabediny et al. 2018). Incorporating FO between the non‐polar tails of phospholipids or using Ω‐3‐rich phospholipids in liposome assembly has been explored. Studies indicate that encapsulating oils within liposomes enhances oxidative stability during storage. However, challenges include the relatively high cost of phospholipids, difficulties in large‐scale production, and low physical stability in complex food matrices (Gulzar and Benjakul 2020).

Some investigations suggest that incorporating nano‐encapsulated FO into certain food products provides superior stability and sensory characteristics compared to food fortified with free FO (Ghorbanzade et al. 2017). Rasti et al. (2012) studied liposome formulations with Ω‐3‐rich fish oils, revealing that PUFAs encapsulated in nanoliposomes (*d* = 50–200 nm) exhibit higher oxidative stability than those encapsulated in larger liposomes (*d* > 200 nm). This difference is attributed to colloidal particle composition, size, and charge variations. Notably, producing liposomes without organic solvents has been shown to protect them from oxidation. Recent studies have also explored encapsulating shrimp oil using nanoliposomes through innovative methods, such as microfluidization and ultrasonication, resulting in nanoliposomes with high encapsulation efficiency and oxidative stability (McClements 2012).

### 6.2 Emulsions and Nano‐Emulsions

Emulsions and nano‐emulsions, defined as small emulsifier‐coated oil droplets dispersed in water, are widely accepted for encapsulating fish oils. Nano‐emulsions, with droplet diameters below 100 nm, offer improved bioavailability and physical stability of Ω‐3 oil formulations. Various homogenization methods, such as high‐shear mixers, colloid mills, high‐pressure valve homogenizers, microfluidizers, and sonicators, are employed for emulsion formation. Researchers are exploring unconventional emulsions, including high‐internal‐phase and Pickering emulsions, to enhance functionality. Studies indicate that nano‐emulsions‐based delivery systems enhance water‐dispersibility, physical stability, and bioavailability of PUFAs in food products (Walker et al. 2015; Walker et al. 2017; Jiang et al. 2020).

Nanoemulsions have emerged as a cutting‐edge encapsulation technology for Ω‐3 FAs, such as those found in FO, due to their superior stability and enhanced bioavailability. These colloidal systems consist of oil droplets dispersed in an aqueous phase, stabilized by emulsifiers, with droplet sizes typically below 100 nm (Walker et al. 2015). The small droplet size significantly increases the surface area, allowing for improved absorption and bioactivity of Ω‐3 FAs while also providing robust protection against oxidative degradation. Various techniques, such as high‐pressure homogenization and ultrasonication, are commonly employed to produce nanoemulsions, ensuring uniform droplet distribution and long‐term stability (Chen et al. 2017). Nanoemulsions also exhibit excellent compatibility with different food matrices, making them ideal for incorporating Ω‐3 FAs into functional foods and beverages without altering sensory characteristics. Additionally, the encapsulation of Ω‐3 FAs in nanoemulsions reduces the unpleasant odor and taste often associated with FO, thereby enhancing consumer acceptance (Ghorbanzade et al. 2017).

### 6.3 Solid Lipid Nanoparticles (SLNs)

SLNs and nanostructured lipid carriers (NLCs), which are structurally similar to nano‐emulsions but with fully or partially crystallized lipid phases, have been developed to enhance stability by slowing down the diffusion of pro‐oxidants. SLNs and NLCs are prepared using methods similar to emulsion creation, with homogenization above the lipid phase melting point and subsequent cooling below the crystallization temperature. Careful formulation is required to prevent the exclusion or accumulation of bioactive compounds during droplet crystallization (Kharat and McClements 2019; Ghanbarzadeh et al. 2019).

SLNs have garnered significant attention as a promising encapsulation method for Ω‐3 FAs, particularly those derived from FO. SLNs are submicron‐sized particles composed of biocompatible and biodegradable lipids that remain solid at both room and body temperatures. This solid matrix effectively protects encapsulated Ω‐3 FAs from oxidative degradation, thereby enhancing their stability and prolonging shelf life. Moreover, SLNs facilitate controlled release and improve the bioavailability of these PUFAs. Recent studies have explored the incorporation of antioxidants, such as quercetin, into SLN formulations to further inhibit lipid oxidation (Azizi et al. 2019; Venugopalan et al. 2021; Tang et al. 2023).

### 6.4 Multiple Emulsions

Multiple emulsions, categorized as water–oil–water (W/O/W) and oil–water–oil (O/W/O) emulsions, offer a more complex structure. W/O/W emulsions, suitable for FO encapsulation, consist of small water droplets within larger oil droplets dispersed in a continuous water phase. These emulsions find applications in the food industry for calorie reduction, fat reduction, flavor masking, controlled release, and protection of sensitive ingredients. The formation involves a two‐step procedure, forming a W/O emulsion followed by further homogenization with a water phase, resulting in fish oils forming an oil phase separated by two aqueous phases (Cournarie et al. 2004; Hwang et al. 2017; Yan et al. 2020).

### 6.5 Microgels

Edible microgels, composed of small particles from food‐grade proteins and/or polysaccharides (Farjami and Madadlou 2017), form a network of physically or chemically crosslinked biopolymer molecules (Timilsena, Vongsvivut, et al. 2019). Typically, Ω‐3 oils are emulsified and incorporated into microgels using various methods, such as injection, phase separation, and molding (Carvalho et al. 2016; Chang and Nickerson 2018). Different combinations of biopolymers, including hydroxypropyl methylcellulose‐maltodextrin and whey protein‐gum arabic, have been explored for encapsulating FO within coacervates (Shishir et al. 2018).

### 6.6 Nanofibers

Nanofibers, comprising long, thin, fibrous materials assembled from food‐grade biopolymers, offer a means to encapsulate and control the release of hydrophobic substances. For example, electrospraying can be used to encapsulate DHA in zein nanofibers, enhancing oxidative stability (Busolo et al. 2019).

### 6.7 Inclusion Complexes

Inclusion complexes involve trapping bioactive molecules within a cyclic oligosaccharide, such as cyclodextrin, forming a molecular inclusion complex. For FO, the non‐polar tails of fatty acids are trapped within the hydrophobic cavity of cyclodextrin, leading to improved oxidative stability. Studies show high encapsulation efficiency for FO within cyclodextrin inclusion complexes (Choi et al. 2010; Paul et al. 2017).

### 6.8 Microencapsulation

Microcapsules containing CSO were formulated utilizing three distinct wall materials: CS gum, CS protein isolate, and a combination of complex organic‐rich droplets with chia protein isolate‐CS gum. The influence of these diverse wall materials on chia seed microcapsules was assessed through Synchrotron‐Fourier transform infrared micro‐spectroscopy (FTIRM). FTIRM analysis facilitated the measurement of lipid fractions within the microcapsules and provided insights into lipid levels. A comparative study encompassing FTIRM chemical images, surface protein‐lipid content, and internal microcapsule structure was conducted. The ratio of CSO to microcapsules exhibited no significant differences across the three types of wall materials. Notably, microcapsules generated through complex coacervation exhibited a content below 2% (w/w), while the other two types of microcapsules exceeded 5% (w/w) (Timilsena, Vongsvivut, et al. 2019). The evaluation of the physicochemical analysis of CSO design micro‐particles was produced using maltodextrin, two layers of sunflower lecithin, either mono or bi, chitosan, and phosphatidylcholine (PC) as a wall material. The physicochemical analysis of powdered chia oil microparticles indicates that Ω‐3 PUFAs (N 89%) have the highest value in all cases and record the highest Deoiled by PC. However, the bi‐layered classifications showed a higher percentage of lasting Ω‐3 and lower peroxide value (PV) than the monolayer system with raw oil. The results indicated that the PV of chia oil microcapsules was excellent when using the modified sunflower lecithin emulsions as a wall material (Julio et al. 2019).

The biosorption of CSO extraction was analyzed using the batch process. This method was affected at 303 K temperature for 1 h and characterized wastewater treatment. This process was also affected using the studies of equilibrium, kinetics, and thermodynamics. The results indicated the maximum efficiency level at pH 2 and at 150 rpm agitation speed. Moreover, the dye removal from the solution ranged precisely to 92%, and the 2nd order of pseudo was perfect. Bio‐sorption capacity was shown to have the highest range of 70.95 mg with mass center at G1, with a heterogeneity parameter (1.2785), respectively (da Silva and de Abreu Pietrobelli 2019). In addition, many researchers prepared the CLA of microcapsules using three different coated materials (DE 10 maltodextrin, whey protein, and gum arabic). The maximum CLA and lipid oxidation value was measured at 0.103–0.429 of water activity (a_w_) for all wall material users. The lowest value of CLA with lipid oxidation was obtained at the a_w_ (0.743) at 35°C for whey protein concentrates. The results concluded that the microcapsules of whey protein indicate low CLA degradation, are more efficient, and have the best particular form, shape, or structure (Duhoranimana et al. 2018; Zhuang et al. 2018). In another research work, microcapsules of CSO were prepared using the spray drying method (Us‐Medina et al. 2018). For spray drying, the emulsion was prepared with various wall materials such as maltodextrin (MD). The oxidative levels of FO microcapsules were estimated using the spray drying method for FO emulsion. FO emulsion contains a solution of cellulose and FO. The size and appearance of the prepared microcapsules were estimated using a laser light microscope and an electron microscope. The results revealed that the PV of microcapsules was higher than that of the FO emulsion (Bannenberg et al. 2017).

Therefore, the oxidative stability of microcapsules was not impaired (Ahmmed et al. 2020). FO is an excellent source of LCPUFAs. FO is less stable against oxidation, an essential function in food industries. Therefore, microcapsules of FO were made using a spray drying method. Cellulose and MD emulsions were used as wall material in the microencapsulation process. This homogenized emulsion was able to control oxidative stability. In this study, the results indicated that the homogenized emulsion is a more stable foam, and there was more injury in the formation of powder capsules due to hydroxypropyl methylcellulose. Therefore, the structural damage of the sample was greater when compared to the stabilization effect. At the end of the results, spray‐dried microcapsule improves FO's oxidative stability and increases FO powder concentration (Shi et al. 2020).

Multilayer microencapsulation was analyzed using chitosan and MD as wall material. This wall material can control the oxidation of lipids in micro‐capsulated FO. All prepared samples were analyzed, and the oxidative stability of the microcapsule and the oxidative response during the 60‐day storage period were found to be positive. PV was used as the primary oxidation material, and *p*‐anisidine value with thiobarbituric acid reactive substance (TBARS) assay was used as a peripheral oxidative compound. The results indicated that the designed sample increased the oxidative stability of micro‐capsules and improved the value of the different parameters (Assadpour and Jafari 2019). Fish oil microcapsule (FOMC) was prepared using spray drying with different wall materials. RSM and the evolutionary algorithm were used to elevate the FOMC. In phase 1, the preparation and characterization of FOMC were measured according to the terms and conditions of spray drying. Moreover, the spray drying terms and conditions were developed as a model to measure the value of an evolutionary algorithm. The highest spray drying FOMC was analyzed in the optimization conditions of inlet temperature 177.2°C, ejector‐jet pump rate 63.9%, and roller pump rate range from 14.04%. Furthermore, the powder yield of spray drying ranged from 8.10% to 79.1%. The results suggest that these terms can be used to manufacture FOMC with spray drying (Timilsena, Vongsvivut, et al. 2019). Furthermore, the spray drying process for preparing FOMC uses MD, casein, and cluster dextrin as wall material. Casein was used along with the wall material to improve the oxidative stability of the FOMC. In addition, this wall material was affected by the final product and enzymatic browning. Additionally, the surface of microcapsules was also affected by using dextrin. The results concluded that FOMC was prepared with dextran in the spray drying process, which affects the microcapsule surface (Xiong, Liu, et al. 2021; de Melo Ramos et al. 2021).

## 7 Functional Food Products

For contemporary individuals, the selection of nutritionally rich and healthful foods poses a challenge for food manufacturers. This research investigates the rheological properties of chia seed (CS) gums, employing dynamic, shear, and thermal analyses. Furthermore, the CS gum is incorporated into the formulation of cookies. Initially, the CS gum sample exhibits distinct peaks in the differential scanning calorimetry (DSC) thermogram. Subsequently, the CS gum undergoes analysis at various temperatures (To, Tp, and Te) corresponding to the endothermic peak (52.8°C, 107.9°C, and 215°C) and exothermic peak (277.7°C, 316.8°C, and 354.9°C). The cookies prepared with CS gum demonstrate the maximum values for diameter, thickness, and spread factor. Moreover, the spread factor of the experimental product exhibited a reduction ranging from 6.83 to 5.99, correlating with an elevated concentration of CS gum. The sensory profile of the cookies demonstrated favorable attributes in terms of color, texture, and mouthfeel, particularly when formulated with 30% CS gum, resulting in an overall acceptability (Punia and Dhull 2019). In the preparation of rabbit meat, varying levels of CS (0%, 10%, and 15%) were employed. The results from the experiments indicated no significant alterations in feed conversion and the percentage of carcass (live weight/live weight gain) across the three CS inclusion levels. Moreover, the integrity of bones (ribs and hips), back limb, front limb, chest (pectoral) muscles, ribs, head, limbs, and skin remained unaffected. The concentration of Ω‐3 and Ω‐6 in the semispinalis muscles and perinephric fat exhibited an increase corresponding to the higher inclusion levels, while straight‐chain organic acid decreased. Notably, the ratio of essential fatty acids in the control group (4.55) was compared with the experimental group (1.03) at a 15% inclusion level (Peiretti and Meineri 2018).

The soluble fiber found in CS is deemed nutritionally rich, particularly owing to its mucilage content, which serves as an excellent source of soluble fiber. This study investigates the impact of chia seed‐derived soluble fiber on the composition of pita bread and its influence on glycemic index modulation. Various analytical techniques, including laser microscopies, X‐ray diffraction examination, differential scanning calorimetry, and scanning electron microscopy, were employed to scrutinize the bread's structure. The augmentation of fibers in all samples resulted in notable alterations in starch structure, evident in the lowest X‐ray diffraction peaks across all crumb samples, accompanied by consistent gelatinization with amorphous lipids. Crust samples exhibited A‐type diffraction and a 48.7% degree of gelatinization. Comparative analysis revealed that the control crust samples ranged from 73.2% to 69.4%, while experimental bread samples ranged from 69.5% to 66.3%. In contrast, the formulated bread's crumb exhibited the highest values (71.8%, 70.0%, and 73.1%). These findings highlight the influence of crumb soluble fiber on glycemic index, gelatinization degree, and the starch structure of the crust (Salgado‐Cruz et al. 2017).

This study focuses on the characterization of an experimental marmalade with potential health implications, incorporating CSO and dietary fiber as functional ingredients. The research aims to enhance carbohydrates and fatty acids in strawberry marmalade, leveraging the abundant presence of PUFAs in CSO. The initial step involves a sensory evaluation of various formulations utilizing stevia powder and sorbitol as active ingredients, with a control treatment employing commercial sugar for comparison. Hedonic scale assessments revealed overall acceptability for sorbitol. Subsequently, the experimental marmalade was developed with 2.5%–5% CSO and sorbitol, with 5% yielding optimal acceptability. This formulation exhibited increased phenolic content (15.4%) and dietary fiber (168%), along with a 48% reduction in caloric content compared to the control group. In the final phase, the experimental product incorporated 1.5% essential fatty acids, resulting in higher viscosity compared to the control group (Özbek et al. 2019). In another study, a fortified cake was prepared with FO microcapsules to enhance PUFAs such as EPA and DHA for health benefits. Different ultrasonication amplitudes were applied to create the FO in milk emulsion, and FO microcapsules were developed using the spray drying method. The stability of encapsulated FO was assessed at refrigerated and ambient temperatures for 32 days. The results indicated that the microencapsulated FO in the cake exhibited superior oxidative stability at refrigerated temperatures. The overall conclusion suggests that FO capsules are suitable for enhancing Ω‐3 in bakery products (Santhanam et al. 2015).

**FIGURE 4:**
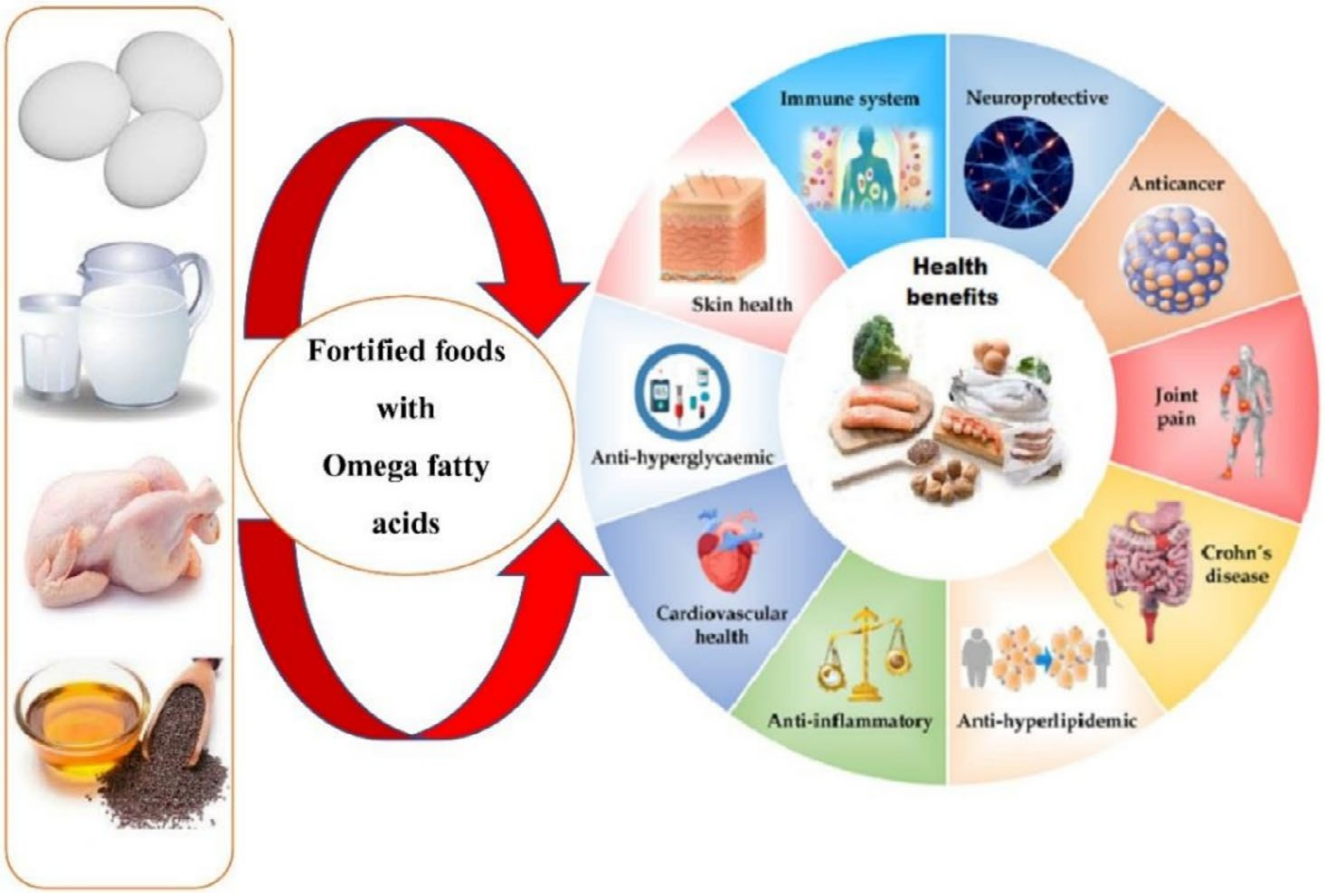
Fortified food with omega fatty acids and health benefits.

Over the past decade, the population's intake of EPA has had unfavorable health effects. Diet supplementation is an excellent way to promote the Ω‐3 series of PUFAs without the basic changes to commonly used food products. This study selected food products to improve Ω‐3 PUFAs in emulsions (30%) and micro‐capsulated powdered (10%) FO. These experimental food products have been used to prevent many diseases. FO supplementation was affecting the palatability of food products. The most involved in instant powder‐milk‐based composition is 18%, which provides 1.8% of DHA and EPA and further lipid content as far as 1.5%, which provides 0.5% DHA and EPA and their products. The high sweetness level is achieved, and the intensity of the flavors, taste, and odor masks the unpleasant fish. Fortified salad oil was not modified significantly throughout the 8 weeks of storage, and concentration was not changed after 12 weeks. Enriched fruit drinks were low pH improved, and their oxidation was reduced. Fortified vegetable and milk juices significantly improved EPA and DHA levels (0.03%), and their palatability was reduced. The results indicate that these enriching food products are overall acceptable and that EPA and DHA improve nutritional values (Kolanowski and Berger 1999; Migliore et al. 2022). FO microcapsules were developed using spray drying and soybean as a wall material. These microcapsules were used in various food products to improve the essential fatty acids for health benefits. The chemical composition of multiple emulsions has excellent encapsulation potential, and their emulsification and formulation techniques were analyzed. A health chemical analysis was performed to determine the protein and oil ratios. As confocal microscopy shows, soy protein managed FO microcapsules, revealing its characteristic odor and oil structure in matrix‐type microcapsules. The emulsification process was a causal factor in the drying, encapsulation efficiency, and the core material used in oil stability (di Giorgio et al. 2019) (Figure 4).

## 8 Health Benefits of Omega‐Enriched Products

Omega‐9 (Ω‐9) fatty acids, prominently represented by OA in olive oil, confer a spectrum of health advantages. OA, a principal Ω‐9 fatty acid, has demonstrated potential in fostering cardiovascular well‐being. Scientific inquiries suggest that a dietary regimen enriched in OA may yield reductions in low‐density lipoprotein (LDL) cholesterol levels, thereby diminishing the susceptibility to cardiovascular pathologies. Furthermore, the anti‐inflammatory attributes inherent to Ω‐9 fatty acids propose a modulatory role in abating chronic inflammatory states, particularly relevant to conditions such as arthritis. OA's association with heightened insulin sensitivity introduces potential therapeutic implications for those contending with diabetes or insulin resistance. The monounsaturated fatty acids characteristic of Ω‐9 contribute substantively to cellular membrane stability, thereby influencing overall cellular functionality. Moreover, the presence of Ω‐9 fatty acids in olive oil manifests supportive effects on skin physiology, influencing moisture retention and elasticity (Djuricic and Calder 2021; Alagawany et al. 2022).

CS is a promising source of essential fatty acids. It was used to measure the effect of ALA on metabolic processes, liver symptoms, and formulate a diet in obese rats. Mice were fed a high‐fat diet using fat, carbohydrate, and fructose in drinking water. CS was supplemented with 5% for 2 months and then 2 months on a high‐fat diet. The results indicated that the high‐fat rats, rats showed reduced body fat, liver dysfunction, and liver and heart inflammation with no change in blood pressure, glucose tolerance, and insulin response. Lipid redistribution through essential fatty acids is rich in CS, stimulating liver and visceral fat and increasing heart rate accumulation. CS stearoyl‐CoA desaturase‐1 was eliminated, and obesity promoted cardiac protection in the liver and heart of mice. PUFAs supplementation was stored in various parts of the rat body, such as the biosynthetic pathway of PUFAs in metabolism, *trans*‐9‐octadecenoic acid in sensitive organs, and vaccenic acid in connective tissue or fat. The results concluded that CS supplemented a good ALA, EPA, and DHA source, which causes lipid distribution and reduces heart diseases (Grancieri et al. 2019; Munda et al. 2022). In another research work, CS is a good source of Ω‐3 fatty acids because many research studies have been done on the effects of CS, which have increased in recent years (Khalid et al. 2023). Therefore, this dietary CS improved dyslipidemia and hepatic steatosis in rats fed for 1–3 weeks. This complex hepatic enzyme activity involves lipid metabolism and sterol regulatory factors binding protein and protein mass levels. Sucrose‐rich diet‐fed rats had improved liver fat, hepatic enzyme activity, and dyslipidemia (Felemban et al. 2021).

The pathophysiological role of plasma levels, inflammation, and oxidative stress leading to tissue damage has been implicated in cardiovascular diseases. This study investigates the Spirulina and FO, which have shown an effect on hypercholesterolemia. The results indicated that the high cholesterol group combined with the spirulina post‐enhanced oxidative stress and inflammation, altering the plasma lipid and antioxidant capacity. Furthermore, the FO group promoted the formation of fatty acids in the arteries and increased plasma fat, which was inversely proportional in the case of spirulina. Additionally, inflammation and oxidative stress were prevented in the case of FO and the Spirulina group. The results indicated that FO and spirulina are more effective against hypercholesterolemia (Muga and Chao 2014; Xiong et al. 2018).

In hemodialysis patients, the effect of FO on fat profile was analyzed using different control trials. This research conducted 209 prospective studies and 13 randomized controlled trials consensus using the selected criteria. When compared with the results of the FO group with the control group, it was found that total cholesterol and triglyceride levels were low from 0.23 to 0.12 mmol/L. Moreover, the effect of FO supplementation (FOS) on high‐density lipoprotein was enhanced (0.20 mmol/L) and did not affect the low‐density lipoprotein. The result of the research concluded that the FOS improved the high‐density lipoprotein and reduced the low‐density lipoprotein, serum triglyceride, and serum cholesterol in hemodialysis patients. FOS is very effective against cardiovascular diseases (Zhu et al. 2014). Furthermore, Xin et al. (2012) reported the influence of FO on chronic heart failure patients, which is still controversial. Human randomized control trials were compared to an experimental group of patients in this research work. Moreover, interleukins 1 and 6 were significantly decreased after a dose of FO in a systemic inflammation patient. Furthermore, there was no significant change in the *p*‐value of vascular cell adhesion protein 1, lymphocyte function‐associated antigen, and high‐sensitivity C‐reactive protein. The result concluded that the impact of FO against TNF alpha and infection and tissue injury was more effective (Lin et al. 2019).

FO plays a significant role in preventing the impact of glucose intolerance, lipid profile, and metabolic pathway through the autonomic nervous system, a large group of proteins, peptides, and stress hormones such as glycogen, corticosteroid, and monoamine neurotransmitters. In this research, FO is consumed to avoid environmental stress, the concentration of adiponectin receptor 1 (AdipoR1) and serum, and in adipose tissue and liver, exert pro‐ and anti‐inflammatory cytokine activity and TNF alpha. The 32‐day‐old male Wistar mice were distributed into four groups: two control and two experimental groups of stressed and non‐stressed mice. None of the mice in this group had different body fat, protein, or weight. The studies showed that the serum corticosterone and basal serum glucose concentrations were improved in stressed‐fed rats compared to the other groups. Therefore, after 15 min of foot shock stress, the levels of corticosterone were not changed in all groups, and after feeding the FO in stressed groups, that inhibited the pro‐ and anti‐inflammatory cytokine activity. The protein concentration was not determined in foot shock stress, but the protein concentration of adiponectin receptor 2 (AdipoR2) was improved in FO diet rats. The concentration of AdipoR1 was similar in all groups. The response of stressed rats was normalized in a group of mice fed with FO. The results concluded that the FO is more effective against disease development through stress (Lima Rocha et al. 2022). In addition, FO is commonly consumed as a nutritional supplement to improve the Ω‐3 fatty acids for health benefits. Nowadays, FO is one of the most commonly used supplements in the general population because it improves EPA and DHA levels and improves the effect of physical health and the 12‐item short form (SF‐12) in adults. This study was performed on the Ω‐3 FAs index as well as on virtual clinical research and assessed EPA and DHA in adult red blood cells. These total fatty acids reduce the risk factors for cardiovascular diseases. In FO adults, these Ω‐3 fatty acids significantly improved mental health with no change in physical health. The result suggests that Ω‐3 fatty acids increase in healthy populations that consume the most (Udani and Ritz 2013).

**FIGURE 5:**
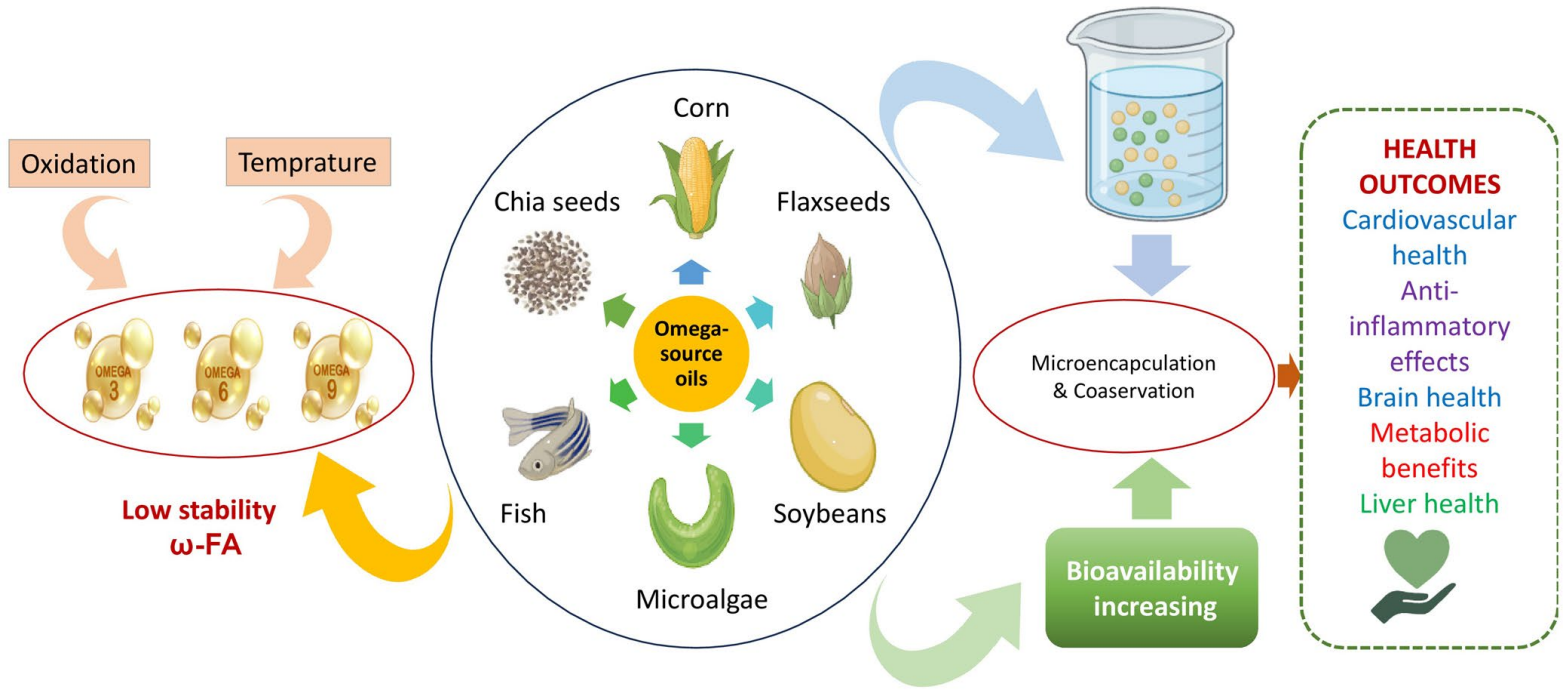
Health benefits of Ω‐3 fatty acids and their mechanism.

The effect of FO on type 2 diabetes mellitus (T_2_ DM) has not been studied. This research work investigated the effect of FO and its low‐fat profile and its role as insulin‐sensitizing agents. In this study, 1‐month‐old UC Davis (UCD)‐T_2_ DM mice were subdivided into three groups for analysis. These mice were fed FO at a rate of 3 g/kg body weight per day, with 1 g/kg body weight per day of EPA. The results showed that when the control group was compared to mice at 16 weeks, the fasting plasma triacylglycerides concentration was significantly decreased in the FO group by 39% (SD‐7), and EPA treatment also reduced the concentration of fasting plasma NEFA by 23% (SD‐5). Furthermore, the concentration of fasting plasma cholesterol was reduced from 22% to 19% (SD‐4) in 16‐week‐old mice. The final results suggest that FO and EPA do not affect T2DM in mice but reduce circulating fat concentration (Parkman et al. 2021). Research on the impact of FO on cardiovascular health, focusing on heart rate variability (HRV) and arterial elasticity, yielded interesting results. The study found that despite no significant effect on traditional cardiovascular measures such as blood pressure and heart rate, FO intake led to a notable decrease in both low and high frequencies by 20.34. This suggested a complex modulation of autonomic regulation. Importantly, the elasticity of large arteries significantly improved by 20.31. Overall, while FO may not distinctly influence conventional cardiovascular metrics, it shows promise in enhancing heart autonomic regulation and arterial elasticity, potentially mitigating cardiovascular risk factors (Chu et al. 2021). However, concerns are being expressed regarding some fatty acids derived from plants like soy isoflavones, particularly when taken as concentrated supplements. According to some data, genistein may stimulate the growth of breast tissue in male mice, but other data point to the opposite outcome. Additionally, research revealed that babies who drank soy formula had higher blood levels of isoflavones than women who took soy supplements and experienced irregular menstruation. Additionally, some human and animal research findings have connected soy isoflavones to goiter (Lokuruka 2010). Moreover, concerned about environmental and climatic degradation caused by the lipid‐producing activities of the animal husbandry industry, to avoid the contradiction between the demand for high‐quality of human nutrition and the strain on resources, and to reduce the health risks caused by saturated fats and trans fats in meat products, new avenues for the future edible lipids—such as research into fat and oil substitutes, the use of biotechnology in lipids, and the value‐added reuse of waste products—should be in full swing. The article, therefore, began with a detailed overview of the known lipids available, understanding their origins and the ways in which they are mostly classified. Secondly, possible trends and potential strategies for dietary lipids for use in future foods are presented. Problems and challenges that may be encountered in the research and subsequent industrialization process (Figure 5).

## 9 Functional Food Aspects/Bioavailability of Omega Fatty Acids

The development and manufacturing of functional foods enhanced with bioactive components is now necessary for a healthy living due to modern dietary practices. Nevertheless, not much research has been done on what happens to many of these bioactive substances in the human gastrointestinal (GI) tract. Recent studies cover the bio‐accessibility of omega‐3 fatty acids (Floros et al. 2022). The INFOGEST protocol has been used to simulate the digestion of various diets and supplements. The chosen samples included meals high in omega‐3 fatty acids—in both free and bound form, such as heat‐treated fish, dietary fish oil supplements, and omega‐3 fatty acid‐enriched eggs that were investigated.

Quantification of Omega fatty acids was done with PV and TBARS, as well as a gas chromatograph with flame ionization detector (GC‐FID) in the digestive process. The findings showed that omega‐3 fatty acids underwent a significant oxidation process, producing primary and secondary oxidation products. Furthermore, it appeared that stomach conditions had the biggest impact on PUFA oxidation during digestion, greatly reducing their bioaccessibility (Nieva‐Echevarría et al. 2020).

It is commonly known that omega‐3 (ω‐3) polyunsaturated fatty acids (PUFAs) are good for human health and can treat illnesses. However, as they have a lot of double bonds, they are vulnerable to environmental factors that lead to oxidative degradation. This is one of the primary things that restricts their use. Enhancing the oxidative stability of ω‐3 PUFAs is being achieved through encapsulation. Many researchers added the latest developments in ω‐3 encapsulation systems, including liquid crystalline, liposomes, microcapsules, and emulsions. The impact of food‐derived biopolymer wall components (proteins, polysaccharides, and esters) on the oxidation stability and encapsulation effectiveness of ω‐3 PUFA encapsulation systems is being examined. Furthermore, the impact of natural antioxidants (polyphenols, carotenoids, flavonoids, plant essential oils, etc.) on the antioxidant improvement of the food‐derived biopolymer‐based ω‐3 PUFA encapsulating system is extensively studied. It has been demonstrated that several ω‐3 PUFA encapsulation technologies can be utilized as supplements in the medical industry. A thorough discussion (Du et al. 2022) of the control and enhancement of the ω‐3 PUFAs encapsulation system on human and animal disorders that are associated with the retina, lung, kidney, central nervous system, and tissue cells is available now.

Initiatives to increase the daily consumption of lipids rich in omega‐3 FAs are being aggressively supported by the scientific community and industry. To increase the stability of edible oils high in PUFA against oxidation, a variety of techniques have been used, such as physical mixing, interesterification, and encapsulation. A tried‐and‐true method for improving the oxidative stability and functional characteristics of oils high in omega‐3 FA is encapsulation. To enhance and stabilize the distribution of omega‐3 FAs in food products, a variety of encapsulating techniques have been developed. The intended use of the encapsulated oil determines which encapsulation technique is best. Furthermore, by encouraging greater absorption of the encapsulated form in the intestinal epithelium, encapsulation improves the bioavailability of omega‐3 FAs (Nieva‐Echevarría et al. 2020; Homroy et al. 2024).

## 10 Conclusions

CS, flax seeds, spirulina, linseeds, sunflower seeds, and fish oil (FO), abundant sources of omega fatty acids, bring many health benefits. These omega fatty acids are vital in maintaining cardiovascular health, liver function, and healthy lipid profiles. Studies have reported their significant potential in reducing inflammatory biomarkers and oxidative stress, thus improving cardiovascular health and reducing the risk of arterial hardening. These omega fatty acids have also been shown to improve mental health as they play a significant role in ameliorating oxidative stress in the body, thus helping reduce neurodegenerative diseases. Omega fatty acids are PUFAs with a significant role in reducing the risk of elevated low‐density lipoproteins and triglycerides and maintaining cholesterol levels at lower values, thus improving blood flow and preventing plaque formation in the arteries. Omega fatty acids, being unsaturated, play a key role in keeping the liver healthy. Omega fatty acids significantly lower the accumulated fat in fatty liver cells and ameliorate conditions like hepatic steatosis. Omega fatty acids protect the membranes of the brain and retina cells. Omega fatty acids maintain healthy distribution and reduce circulating fat levels in blood plasma. Omega fatty acids have shown the most significant role in reducing systemic inflammation and autonomic regulation in heart diseases and are also known to reduce environmental stress factors. Ω‐3 fatty acids prevent the development of RA, improve muscle metabolism, and limit muscle atrophy in obese and insulin‐resistant subjects. Omega fatty acids bring countless health benefits and have ameliorative effects on many pathophysiological effects of various diseases by reducing their complexity and adverse effects. On the other hand, excessive use can lead to goiter, inflammation, and other abnormalities.

## Author Contributions

Conceptualization: M.A.R. Editing: M.U. Validation: F.A.‐A. and H.K. Resources: M.A.R. and M.F.R. Data curation: M.A.R. Writing – original draft preparation: M.A.R. Writing – review and editing: M.A.R., R.C.M. and F.A.A. Visualization: M.A.R. and H.K. Supervision: M.A.R. Funding: R.C.M. and E.Z. All authors have read and agreed to the published version of the manuscript.

## Ethics Statement

The authors have nothing to report.

## Consent

The authors have nothing to report.

## Conflicts of Interest

The authors declare no conflicts of interest.

## Data Availability

Data are available from the corresponding author upon request.
